# Systemic cascades on inhomogeneous random financial networks

**DOI:** 10.1007/s11579-022-00315-7

**Published:** 2023-01-10

**Authors:** T. R. Hurd

**Affiliations:** Mathematics and Statistics, McMaster University, 1280 Main St. West, Hamilton, ON L8S 4L8 Canada

**Keywords:** Systemic risk, Banking network, Random financial network, Cascade, Interbank exposure, Funding liquidity, Insolvency, Locally tree-like, 05C80, 91B74, 91G40, 91G50

## Abstract

This article presents a model of the financial system as an inhomogeneous random financial network (IRFN) with *N* nodes that represent different types of institutions such as banks or funds and directed weighted edges that signify counterparty relationships between nodes. The onset of a systemic crisis is triggered by a large exogenous shock to banks’ balance sheets. Their behavioural response is modelled by a cascade mechanism that tracks the propagation of damaging shocks and possible amplification of the crisis, and leads the system to a cascade equilibrium. The mathematical properties of the stochastic framework are investigated for the first time in a generalization of the Eisenberg–Noe solvency cascade mechanism that accounts for fractional bankruptcy charges. New results include verification of a “tree independent cascade property” of the solvency cascade mechanism, and culminate in an explicit recursive stochastic solvency cascade mapping conjectured to hold in the limit as the number of banks *N* goes to infinity. It is shown how this cascade mapping can be computed numerically, leading to a rich picture of the systemic crisis as it evolves toward the cascade equilibrium.

## Introduction

Systemic risk, the risk of large scale failure of the financial system as defined for example in Schwarcz [34], has long been viewed by Kaufman [31] and others as cascades of diverse types of contagious shocks including funding liquidity shocks, solvency shocks and indirect shocks through asset fire sales and bank panics. These shocks are propagated through a diverse network of financial institutions, including banks, funds and other entities. This diversity of shocks and types of institutions points to the need for highly inhomogeneous models of the financial system. Moreover, incompleteness of information in financial systems implies our models need to be stochastic.

The first aim of this article is to introduce *inhomogeneous random financial networks (IRFNs)* as a stochastic modelling framework for the balance sheets of a large, inhomogeneous collection of financial institutions. At the core of financial network models, e.g. Gai and Kapadia [21], Amini et al. [4], Detering et al. [17] and Hurd [27], is the choice of a random graph distribution for the “skeleton” of *N* banks connected by directed edges that represent interbank exposures pointing from debtor to creditor bank. In those models, the skeleton graph is taken to be a *directed configuration graph*, an important generalization of the Erdös-Renyi random graph. Amini and Minca [3] and Detering et al. [17] have studied an alternative family called *directed inhomogeneous random graphs (DIRGs)* that better capture the diversity of bank sizes, connectivity and types. The IRFN framework proposed here continues in this vein by assuming the skeleton graph to be a DIRG.

Given a suitable stochastic network model for banks with their balance sheets, another critical issue for systemic risk is to determine how the default of any bank leads to solvency shocks that hit other banks, creating cascades that may amplify within the financial system. The above cited articles follow a common approach by investigating generic vulnerabilities of banking systems when shocks derive from an assumed mechanistic behaviour of banks. Such a *solvency cascade mechanism* is often analogous to the payment clearing conditions arising in the EN 2001 model, the paradigmatic network model introduced by Eisenberg and Noe [20]. The aim is then to characterize the crisis dynamics and crisis equilibrium that eventually arise through iteration of the *cascade mapping* that encodes this cascade mechanism. For analytical tractability, most contributions to the random financial network literature adopt an over simple cascade mechanism such that the shocks generated by an insolvent bank are a fixed loss-given-default fraction on its interbank debt. This type of discontinuous “hard threshold” assumption excludes the continuous clearing mechanism of the EN 2001 model that assumes zero bankruptcy costs, and many of its natural generalizations. A second goal of the present article is to overcome this limitation, and analyze systemic risk when the recovery fraction of a defaulted bank depends on its degree of insolvency and may take any of a continuum of values. Accounting for such “soft threshold” features of transmitted shocks is a substantial technical extension of systemic risk theory that encompasses more realistic families of cascade mappings on any random financial network.

IRFN models for a finite number *N* of banks are agent based models that can be explored by pure simulation. Alternatively, as for directed configuration skeletons, sequences of DIRGs parametrized by increasing size *N* have an important property often called *locally tree-like (LT)*. This property leads to important analytical results because the random graph sequence is convergent as $N\rightarrow \infty$ to a connected Galton-Watson random tree. This means that the density of cycles of ant fixed length *k* in the random skeleton graph goes to zero as *N* goes to infinity. Amini et al. [4] and Detering et al. [17] have used a theorem of Wormald [39] to prove that certain solvency cascade mechanisms on LT sequences of random skeleton graphs have large *N* asymptotics of the cascade equilibrium that are determined by an average default probability defined as a fixed point of a scalar-valued function. Although the Wormald theorem is powerful, its combinatorial nature means it cannot easily handle the continuum of bank solvency levels that arises from the EN 2001 cascade mechanism. The large *N* asymptotic results continue to hold in a much broader context.

We shall identify an alternative *tree independent cascade property* that ensures that the iterated cascade mapping can be solved recursively when the underlying skeleton graph is a finite tree. Conjecturally, whenever a tree independent cascade mechanism acts on a locally tree-like random skeleton, there will be an explicit recursive stochastic representation of the cascade mapping in the large *N* limit. Under this conjecture, iterations of the cascade mapping converge to a collection of probability distributions representing a *cascade equilibrium* or fixed point of the cascade mapping. It has been observed in Melnik et al. [32] that such fixed points and related asymptotic formulas often provide an “unreasonably effective approximation” of finite sized systems studied by simulation. Moreover, subtle properties of the modelled system, notably its resilience to large cascades, can be understood through sensitivity analysis of the large *N* fixed point.

In summary, the main contributions of this article are:

1. Introduction of the *inhomogeneous random financial network (IRFN) framework*, closely related to the modelling framework of Amini and Minca [3] and Detering et al. [17], that provides a flexible and scalable architecture for modelling many of the complex network characteristics thought to be relevant to systemic risk, specifically random node types, balance sheets and exposures.
2. We present for the first time a stochastic cascade analysis for an economically important family of solvency models with *partial fractional recovery* of defaulted interbank assets, that includes the EN 2001 framework and an extension introduced by Glasserman and Young [24]. Techniques are developed for insolvency level indicators of banks that take a continuum of values in [0, 1], not simply the binary values $\{0,1\}$.
3. We formulate and prove a tree independent cascade property for this family of solvency cascade mechanisms. It is conjectured that cascade mechanisms with this property acting on a “locally tree-like” random skeleton, should have analytically tractable large *N* asymptotics.
4. Following up on the previous point, the large *N* asymptotics for solvency cascades in IRFN models is developed, yielding explicit computable recursive probabilistic formulas for the *cascade equilibrium*.
5. Finally, for the first time, we exhibit a numerical exploration of the large *N* cascade equilibrium in models with a soft threshold solvency mechanism.

The IRFN construction, in common with Amini and Minca [3] and Detering et al. [17], provides two specific benefits compared to the configuration graph RFN constructions of Gai and Kapadia [21] and Amini et al. [4] and others. First, unlike the node degree, bank types represent financial characteristics that do change only slowly over time, if at all. Allowing an arbitrary finite number of bank types can encode an unlimited range of node characteristics. Second, bank type makes good financial sense as a parsimonious conditioning random variable. Thus, we shall assume that random balance sheets and exposures are independent, conditionally on node types.

In addition to the works cited above, the present article also relates to an impressive amount of systemic risk research, for which extensive list of references are provided in reviews by Benoit et al. [5], Capponi [11] and Jackson and Pernoud [30]. To list some of the important recent work, Craig and von Peter [16] and Acemoglu et al. [1] follow up pioneering work by Allen and Gale [2] on the systemic effect of network topology. Approaches to bank resolution, meaning the treatment of the recovery value for defaulted banks, is an input to this article that has been studied from many points of view, including Rogers and Veraart [33], Chari and Kehoe [13], Weber and Weske [37] and Bernard et al. [6]. Other channels of systemic contagion have been studied: Duarte and Eisenbach [19], Capponi and Larsson [12], Cont and Schaanning [15] and Bichuch and Feinstein [7] develop asset fire sales; Gai and Kapadia [22] and Heider et al. [25] focus on funding liquidity contagion; Bookstaber et al. [9] provides an agent-based model of multiple channels. Regulatory challenges and tools in systemic risk have been reviewed in Galati and Moessner [23].

This article is organized as follows. Section 2 generalizes the EN 2001 solvency cascade to include a flexible family of default mechanisms. It then defines and proves the tree independent cascade property of such solvency cascade mechanisms. Section 3 introduces inhomogeneous random financial networks (IRFNs) and explores the large *N* analytical properties of the degree and balance sheet distributions. It proves the first step of the stochastic solvency cascade mapping in the $N=\infty$ limit for a single node type, and then provides a non-rigorous derivation of this argument extended to multiple node types and valid for any time step. Section 4 explores data, calibration and computation issues that arise when the IRFN cascade method is applied to a real world network. Section 5 presents a numerical implementation of the stochastic cascade mapping in a class of IRFN models with fractional bankruptcy costs. Finally, a concluding section discusses next steps for better understanding systemic risk in inhomogeneous random financial networks.

**Notation: ** For a positive integer *N*, [*N*] denotes the set of node labels $\{1,2,\dots , N\}$. Random variables *X* have cumulative distribution function (CDF), probability density function (PDF) and characteristic function (CF) denoted by $F_X, \rho _X=F_X',$ and $\hat{f}_X$ respectively. The $L^2$ norm of a function *f*(*x*) on a domain *D* is defined to be $\left\Vert f\right\Vert _{L^2(D)}=\int _D|f(x)|^2\ \textrm{d}x$. For any event *A*, $\textbf{1}(A)$ denotes the indicator random variable that takes binary values in $\{0,1\}$. Landau’s “big O” notation $f^{(N)}=O(N^\alpha )$ for some $\alpha \in \mathbb {R}$ is used for a sequence $f^{(N)}, N=1,2,\dots$ to mean that $f^{(N)}N^{-\alpha }$ is bounded by a constant as $N\rightarrow \infty$.

## Solvency cascades with bankruptcy costs

The financial system at any time will be a network of *N* banks labelled by $v\in [N]$. Each bank *v* will be connected to another bank *w* by a directed edge labelled by $(vw)\in [N]\times [N]$ whenever there is a significant interbank debt exposure of *w* to *v*, i.e. when *v* owes *w* a significant amount. The resultant directed random graph of credit relationships is called the *skeleton* of the network.

*Cascade mechanisms (CMs)* encode the stylized behaviours that banks follow when a financial crisis has been triggered. These non-linear behaviours reflect assumptions that in a crisis, banks replace “business as usual” strategies by emergency or remedial actions, and sometimes in extreme circumstances are taken over by a *system regulator*. We assume healthy banks “do nothing but wait and see” during a crisis, while weak banks’ behaviour is severely constrained by the regulatory structure. From a systemic perspective, cascades can arise when weak banks’ behaviour negatively impacts other banks. Hurd [27, Chapter 2] provides an overview of some of the important cascade channels that should be included in models of systemic risk. For example, *funding liquidity cascades* arise as banks experience withdrawals by depositors or wholesale lenders that lead them to tighten lending to other banks in the system. The focus of the present article is on *solvency cascades* generated by bank defaults. Defaulted banks transmit shocks to counterparties along edges. Since default shock transmission is mathematically similar to funding liquidity contagion, the cascade mechanisms proposed in this section have broad implications on systemic risk.

### Balance sheets and solvency

The treatment of solvency cascades presented here follows the discussion of Hurd [27, Section 2.1], starting with balance sheets of banks viewed at the coarse-grained resolution shown in Table 1.

**Table 1:** A stylized bank balance sheet

Assets	Liabilities
Interbank assets $\bar{\textrm{Z}}$	Interbank debt $\bar{\textrm{X}}$
External assets $\bar{\textrm{Y}}$	External debt $\bar{\textrm{D}}$
	Equity $\bar{\textrm{E}},\bar{\Delta }$

Prior to the onset of the crisis, a bank *v* has a balance sheet that consists of assets and liabilities $\bar{\textrm{B}}_v:=[\bar{\textrm{Z}}_v,\bar{\textrm{Y}}_v,\bar{\textrm{X}}_v,\bar{\textrm{D}}_v, \bar{\textrm{E}}_v]$ labelled by bars, that represent the *notional aggregated values* of contracts (also called *book values* or *face values*), valued as if all banks are solvent. Assets (loans and securities) and liabilities (debts and equity) are separated into internal and external quantities depending on whether the counterparty is a bank or not. The internal assets $\bar{\textrm{Z}}_v$ and liabilities $\bar{\textrm{X}}_v$ of bank *v* can be disaggregated into the collection of notional exposures $\bar{\Omega }_{vw}$ to its counterparties *w*. Banks and institutions that are not part of the system under analysis are deemed to be part of the exterior, and their exposures are included as part of the external debts and assets. Finally, only a single category of external assets is considered.

#### Definition 1

The *total notional value of assets* $\overline{\textrm{TA}}_v$ of bank *v* prior to the crisis consists of the *internal assets* ${\bar{\textrm{Z}}}_v$ and the *external assets* $\bar{\textrm{Y}}_v$. The *total notional value of liabilities* $\overline{\textrm{TL}}_v$ of the bank consists of the *internal debt* ${\bar{\textrm{X}}}_v$, the *external debt* ${\bar{\textrm{D}}}_v$ and the bank’s *equity* ${\bar{\textrm{E}}}_v$. The *exposure* of bank *w* to bank *v* is denoted by $\bar{\Omega }_{vw}$. All components of $\bar{\textrm{B}}$ and $\bar{\Omega }$ are non-negative, and the *accounting identities* are satisfied:

1 $$\begin{aligned}{} & {} {\overline{\textrm{Z}}}_v=\sum _w \bar{\Omega }_{wv}, \quad {\bar{\textrm{X}}}_v=\sum _w\bar{\Omega }_{vw},\quad \sum _v {\bar{\textrm{Z}}}_v=\sum _v {\bar{\textrm{X}}}_v,\quad \bar{\Omega }_{vv}=0\ ,\nonumber \\{} & {} \quad \overline{\textrm{TA}}_v:={\bar{\textrm{Z}}}_v+\bar{\textrm{Y}}_v={\bar{\textrm{X}}}_v+{\bar{\textrm{D}}}_v+{\bar{\textrm{E}}}_v=: \overline{\textrm{TL}}_v\ . \end{aligned}$$

The full notional balance sheet $\bar{\textrm{B}}_v$ of bank *v* can be reconstructed from the collection of variables $\bar{\Omega }_{vw}$ and $\bar{\textrm{Y}}_v, {\bar{\textrm{E}}}_v$.

Notional values are considered to be applicable to banks that are compliant with all regulatory constraints, prior to the financial crisis. A shock to the system that is sufficient to trigger a systemic risk event or crisis will be modelled as a large instantaneous change to the collection of balance sheets.

#### Definition 2

A *crisis trigger* at a moment in time $t=0$, occurs when a shock $\delta \textrm{B}=[\delta \textrm{Y}, {\delta \textrm{E}}]$ to the balance sheets is sufficiently severe to put some banks into a stressed state where not all of their post-trigger balance sheet entries $\textrm{B}^{(0)}:=\bar{\textrm{B}}+\delta \textrm{B}$ are positive. For simplicity in this article we assume interbank debts are not directly impacted by the trigger, that is $\delta \Omega =0,\Omega ^{(0)}=\bar{\Omega }$.

To maintain the convention that balance sheet entries are never negative, we introduce the *solvency buffer* $\Delta ^{(0)}_v:=\bar{\textrm{E}}_v+\delta \textrm{E}_v$ that may now be negative, in which case the bank is said to be *insolvent* or, equivalently, *bankrupt*. Superscripts (*n*) always indicate the state of a variable at the end of day *n*. In our general systemic risk modelling paradigm, the *cascade* that follows the crisis trigger will be viewed at the end of each day $n\ge 0$ as the day-to-day dynamics for the collection of balance sheets $\textrm{B}^{(n)}_v$ of the entire system as the system tries to *resolve* these insolvent banks.

### Solvency cascade mechanisms

We now want to study how the banks in a network will respond when a crisis trigger event on day $n=0$ leaves some banks insolvent. We consider a class of partial recovery models extending EN 2001, the clearing model for defaulted debt of Eisenberg and Noe [20], to account for bankruptcy costs paid when a firm defaults. To address bankruptcy costs and the potential for dangerous contagion amplification, Rogers and Veraart [33] and Weber and Weske [37] extend the EN 2001 model by assuming that bankruptcy costs given default are dependent on both the endowment $\textrm{Y}$ and the value of interbank assets $\textrm{Z}$. Their models typically have *hard thresholds*, meaning the recovery value is discontinuous at the solvency threshold, creating an effectively infinite shock amplification effect. An extreme version of this assumption often studied in the systemic risk literature, for example Gai and Kapadia [21], is that there is zero recovery on all defaulted interbank debt. In contrast to such models with hard thresholds, here we assume the recovery fraction on defaulted interbank debt is an increasing, possibly continuous, function of the balance sheet ratio $\Delta /\textrm{X}$.

#### Assumption 1

(Fractional Recovery) External debt $\textrm{D}$ is senior to interbank debt $\textrm{X}$ and all interbank debt is of equal seniority. The recovery on interbank debt $\textrm{X}_v$ from any insolvent bank *v* is fairly distributed amongst creditors by the formula

2 $$\begin{aligned} \bar{\textrm{X}}_v\ h_v\left( \frac{\Delta _v}{\bar{\textrm{X}}_v}\right) \end{aligned}$$

Here the *recovery function* $h_v(x):\mathbb {R}\rightarrow [0,1]$ is upper semi-continuous, monotonically increasing and has $h_v(x)=1$ for $x\ge 0$.

Typical forms for the recovery function $h_v$ include:

1. $h_0(x)=\textbf{1}(x\ge 0)$: This is the zero-recovery assumption of Gai and Kapadia [21].
2. $h_1(x)=\min \bigl (1,\max (x+1,0)\bigr )$: This is the case of zero bankruptcy costs assumed by Eisenberg and Noe [20].
3. $h_\lambda (x)=\min \bigl (1,\max (x/\lambda +1,0)\bigr )$ for a fixed parameter $\lambda \in (0,1)$. This case was introduced by Glasserman and Young [24] and follows from the assumption that total bankruptcy costs for a defaulted bank are proportional to the negative part of its solvency buffer.

We point out that methods to study the zero recovery model with $h_0(x)$ need only distinguish solvent and insolvent banks, whereas the general case with $h_\lambda (x), \lambda >0$ discussed here must account for a continuum of solvency levels.

It is shown in Hurd [27, Section 2.1.2] that under the fractional recovery assumption 2, the cascade mechanism has a reduced form in terms of the solvency buffers. For each time step $n> 0$ of the solvency cascade, the solvency buffers satisfy a recursive system of equations

3 $$\begin{aligned} S^{(n)}_{wv}:= & {} \bar{\Omega }_{wv} \left( 1-h_w(\Delta ^{(n)}_w/\bar{\textrm{X}}_w)\right) \ , \end{aligned}$$

4 $$\begin{aligned} \Delta ^{(n+1)}_v= & {} \Delta ^{(0)}_v-\sum _{w\ne v} S^{(n)}_{wv} \end{aligned}$$

that depends only on the initial collection of random variables $\{\Delta ^{(0)}_v,\bar{\Omega }_{wv}\}$. These recursive equations specify how over time each insolvent bank *w* sends a growing shock $S^{(n)}_{wv}$ that impacts the solvency buffers of their creditors *v*.

### Tree independent cascade property

A connected directed graph $\mathcal {G}$ with nodes [*N*] and directed edges $\mathcal {E}$ will be called a directed tree if its undirected counterpart is a tree. In this section, we consider only such directed trees. The components of a directed tree have a natural partial order induced by the direction of links. As we now explore, the solvency cascade mechanism just defined generates only “downstream” shocks $S_{wv}$ that flow from *w* to its creditors *v*. It follows that the solvency cascade mechanism has a specific *tree independent cascade property*: this new concept captures the notion that if $\mathcal {G}$ is a directed tree, then the different shocks $S^{(n)}_{wv}$ hitting any node *v* from different debtors *w* are dependent on mutually disjoint collections of balance sheet variables. This property has important implications for the statistical dependencies that arise for the solvency cascade on random networks.

Every node and directed edge of the directed connected tree $\mathcal {G}=[N]\cup \mathcal {E}$ is connected to a fixed node *w* by a unique path, whose final edge is either into or out of *w*. Thus, for each bank $u\in [N]$ and edge $(wv)\in \mathcal {E}$ we can define natural subsets:

1. $\mathcal {M}^-_u$: the subset of $\mathcal {G}$ not including *u* whose elements are each connected to *u* by a path whose final edge is directed into *u*.
2. $\mathcal {M}^-_{w\backslash v}$: the subset of $\mathcal {G}$ whose elements are each connected to *w* by a path not including (*wv*) .

Note there is a disjoint union for each $u\in [N]$:

5 $$\begin{aligned} \mathcal {M}^-_u=\cup _{w:(wu)\in \mathcal {E}}\left( \mathcal {M}^-_{w\backslash u}\cup \{(wu)\} \right) \ . \end{aligned}$$

#### Definition 3

A solvency cascade mechanism is said to have the *tree independent cascade property* if whenever the interbank edges form a connected directed tree, then for all $n\ge 0$ and $(wv)\in \mathcal {E}$ the transmitted shocks $S^{(n)}_{wv}$ depend only on balance sheet variables $\Delta ,\Omega$ taken from $\mathcal {M}^-_{w\backslash v}\cup \{(wv)\}$.

The following proposition implies the solvency cascade mechanism has the tree independent cascade property.

#### Proposition 1

(Tree independent property of the solvency cascade mechanism) Consider the solvency model when the skeleton $\mathcal {G}=[N]\cup \mathcal {E}$ is a connected directed tree. Then the solvency cascade defined by (3), (4) is such that for all $n\ge 0$ and $(wv)\in \mathcal {E}$,

1. $\Delta ^{(n)}_{v}$ depends only on balance sheet variables taken from $\mathcal {M}^-_v\cup \{v\}$.
2. $S^{(n)}_{wv}$ depends only on balance sheet variables taken from $\mathcal {M}^-_{w\backslash v}\cup \{(wv)\}$.

#### Proof of Proposition 1

Assume inductively that $\Delta ^{(n)}_{v}$ depends only on $\mathcal {M}^-_v\cup {\{v\}}$ variables for some $n=k$ and all *v*. Then, using (3), (4) we find

1. for any $(wv)\in \mathcal {E}$, $S^{(k)}_{wv}=\bar{\Omega }_{wv} (1-h_w(\Delta ^{(k)}_{w}/(\bar{X}_{w\backslash v}+\bar{\Omega }_{wv}))$ depends on $\mathcal {M}^-_{w\backslash v}\cup \{(wv)\}$ variables.
2. The sum over shocks in $\Delta ^{(k+1)}_{v}=\Delta ^{(0)}_{v}-\sum _{w\ne v} S^{(k)}_{wv}$ includes only shocks from edges $(wv)\in \mathcal {E}$ that depend on $\mathcal {M}^-_{w\backslash v}\cup \{(wv)\}\subset \mathcal {M}^-_v$ variables.
3. By (5), $\Delta ^{(k+1)}_{v}$ depends on $\mathcal {M}^-_v\cup {\{v\}}$ variables.

Finally, note also that the induction step is true for $n=0$. These facts are enough to complete the proof by induction on *n* of the statement for all $n\ge 0$. $\square$

#### Remark 1

The tree independent property of the solvency cascade mechanism, combined with (5), implies that conditioned on the skeleton being a connected directed tree $\mathcal {G}=[N]\cup \mathcal {E}$, the sum of shocks $\sum _{w:(wv)\in \mathcal {E}} S^{(n)}_{wv}$ hitting any bank *v* for any $n\ge 0$ is a sum of independent random variables.

## Solvency cascades on IRFNs

We now introduce the *inhomogeneous random financial network* (IRFN) as an extension of the random network approach to systemic risk. An IRFN specifies a random network of banks at a moment in time, with balance sheets as described in the previous section. The framework has two levels of structure. The primary *skeleton graph* denotes the directed random graph on *N* banks labelled by nodes $v\in [N]$ where each directed edge labelled by $(vw)\in [N]\times [N]$ signifies a significant exposure of bank *w* to bank *v*. The secondary *balance sheet* layer specifies the balance sheet random variables of banks, representing potential interbank exposures, conditioned on knowledge of the skeleton graph.

### IRFN structure

Inhomogeneity in the IRFN model arises through classifying banks by a finite number *M* of types. The types $(T_v)_{v\in [N]}$ specify the kinds of financial institutions in the model. Furthermore, they are assumed to completely determine the dependence structure of the balance sheet and exposure random variables. The IRFN framework is quite flexible: exactly the same structure has been introduced to infectious disease modelling in Hurd [26].

The skeleton graph is modelled as a directed inhomogeneous random graph (DIRG), generalizing Erdös-Renyi random graphs, in which directed edges are drawn independently between ordered pairs of banks, not with equal likelihood but with likelihood that depends on the bank types. This class, also called the *stochastic block model*, has its origins in Chung and Lu [14] and Britton et al. [10] and has been studied in generality in Bollobáas et al. [8] and the textbook van der Hofstad [35]. This class of random graph has been applied to systemic risk by Amini and Minca [3] and Detering et al. [18]. The DIRG structure arises by the assumption that directed edge indicators are Bernoulli random variables $I_{vw}$ defined for ordered pairs of banks (*v*, *w*), that are independent conditioned on the assignment of bank types.

#### Assumption 2

(Skeleton Graph) The skeleton graph of an IRFN is a directed inhomogeneous random graph $\mathcal {G}=[N]\cup \mathcal {E}\in \textrm{DIRG}(\mathbb {P},\kappa ,N)$ with *N* nodes labelled by $v\in [N]$, defined by two collections of random variables $T_v, v\in [N]$ and $I_{vw}, v,w\in [N]$.

1. Nodes: Each node of $\mathcal {G}$ represents a bank, and has type $T_{v}\in [M]$ drawn independently with probability $\mathbb {P}(T)$ from a list of *bank types* [*M*] of cardinality *M*.
2. Edges: Directed edges of $\mathcal {G}$ represent the non-zero entries of the *incidence matrix* *I*. For each pair $v\ne w\in [N]$, the entry $I_{vw}$ is the indicator for *w* to be exposed to *v*, which is to say that *v* has borrowed from *w*. Conditioned on the type vector $(T_v)_{v\in [N]}$, the collection of edge indicators $I_{vw}$ is an independent family of Bernoulli random variables with probabilities
  6 $$\begin{aligned} \mathbb {P}[I_{vw}=1\mid T_v=T,T_w=T']=(N-1)^{-1}\kappa (T,T')\textbf{1}(v\ne w)\ . \end{aligned}$$

Here the type-probabilities sum to one $\sum _{T\in [M]}\mathbb {P}(T)=1$. The *probability mapping kernel*, $\kappa :[M]^2\rightarrow [0,\infty )$, determines the likelihood that two banks *v*, *w* of the given types have an exposure edge from *v* to *w*. The assumed *N* dependence is necessary to assure sparseness of the graph for large *N*: for consistency we require that $N-1\ge \max _{T,T'}\kappa (T,T')$.

The initial balance sheets for all banks are derivable from a collection of multivariate random variables that are conditionally independent depending on the information of types.

#### Assumption 3

(Balance Sheets and Exposures) The secondary layer of an IRFN, the collection of initial balance sheets and potential exposures $\textrm{B}^{(0)}_v,\bar{\Omega }_{vw}$ at step $n=0$, are mutually independent random variables, also independent of $I=(I_{vw})$, conditioned on the vector of bank types $(T_v)_{v\in [N]}$.

1. For each bank *v*, the marginal CDF of $\textrm{B}^{(0)}_v=[\textrm{Y}^{(0)}_v,\Delta ^{(0)}_v]$ conditioned on the type vector is an increasing function of $\textbf{x}=(x_1,x_2)\in \mathbb {R}_+\times \mathbb {R}$ taking values in [0, 1] and depending only on $T_v\in [M]$:
  7 $$\begin{aligned} F_\textrm{B}(x_1,x_2\mid T):=\mathbb {P}[\textrm{Y}^{(0)}_v\le x_1,\Delta ^{(0)}_v\le x_2 \mid T_v=T]\ .\end{aligned}$$
  Note that $\textrm{Y}_v^{(0)}$ is a positive random variable whereas the solvency buffer $\Delta ^{(0)}_v$ may be negative. The initially insolvent banks are those with $\Delta ^{(0)}_v<0$.
2. For each edge *vw*, the marginal CDF of ${\bar{\Omega }}_{vw}$ conditioned on the type vector is an increasing function on $\mathbb {R}_+=[0,\infty )$ depending only on $T_v,T_w\in [M]$:
  8 $$\begin{aligned} F_{\Omega }(x\mid T, T'):=\mathbb {P}[\bar{\Omega }_{vw}\le x\mid T=T_v,T'=T_w]\ ,\end{aligned}$$
  such that
  $$\begin{aligned} F_{\Omega }(0\mid T, T')=0,\quad \lim _{x\rightarrow \infty }F_{\Omega }(x\mid T, T')=1\ . \end{aligned}$$

In summary, a finite IRFN representing the system after a crisis trigger amounts to a collection of random variables $(T,I,\textrm{B}^{(0)},\bar{\Omega })$ satisfying Assumptions 1 and 2. Note that any potential exposure $\bar{\Omega }_{vw}$ will be an actual exposure if and only if $(vw)\in \mathcal {E}$, i.e. if $I_{vw}=1$. Note also that the solvency cascades of Sect. 2.2 depend only on the reduced set of variables not including $\{\textrm{Y}^{(0)}_v\}$.

### Analytics of IRFN models

The IRFN framework just introduced specifies the joint distributions of the random variables $T,I,\mathrm{B^{(0)}},\bar{\Omega }^{(0)}$, thereby providing a compact stochastic representation of a given real world network of *N* banks at the moment a financial crisis is triggered. The same distributional data defines a sequence of random networks with varying *N*, and the aim of this section is to investigate the analytical properties of this sequence in the limit $N\rightarrow \infty$. The results we find are important consequences of the locally tree-like property of the DIRG skeleton, meaning the property that the density of cycles (closed loops) of any fixed length goes to zero in the limit.

In the following, we shall make repeated use of the classical Lévy Continuity Theorem (LCT). This theorem, proved in Williams [38], considers a sequence of random variables $X^{1},X^2,\dots$ such that the corresponding sequence of characteristic functions converges pointwise to a function $\phi$. The LCT then states that this sequence converges in distribution to a random variable *X* if and only if the function $\phi$ is a characteristic function. Moreover, under this condition, $\phi$ is necessarily continuous and the characteristic function of *X*.

#### Degree distribution of the skeleton graph

The distribution of the number of counterparties of nodes in DIRGs, in other words their in- and out-degrees, has a natural Poisson mixture structure in the large *N* limit. To show this, permutation symmetry amongst the nodes implies one only needs to consider bank 1 with arbitrary type $T_1=T$, whose in/out degree is defined as the pair $(\textrm{d}_1^-,\textrm{d}_1^+)=\sum _{w=2}^N (I_{w1},I_{1w})$, a sum of conditionally IID (independent and identically distributed) bivariate Bernoulli random variables. Here, each summand has the identical bivariate conditional characteristic function (CF)

9 $$\begin{aligned} \mathbb {E}^{(N)}[e^{ik_1I_{w1}}e^{ik_2I_{1w}}\mid T_1=T]= & {} \sum _{T'\in [M]}\mathbb {P}(T')\left( 1+(N-1)^{-1}\kappa (T',T)(e^{ik_1}-1)\right) \nonumber \\{} & {} \times \left( 1+(N-1)^{-1}\kappa (T,T')(e^{ik_2}-1)\right) \ . \end{aligned}$$

The conditional CF of $(\textrm{d}_1^-,\textrm{d}_1^+)$ is the $N-1$ power of this function:

10 $$\begin{aligned}{} & {} \mathbb {E}^{(N)}[e^{ik_1\textrm{d}^-_{1}+ik_2\textrm{d}^+_{1}}\mid T]\nonumber \\{} & {} \quad =\left[ 1+\frac{1}{N-1}\sum _{T'}\mathbb {P}(T')\left( \kappa (T',T)(e^{ik_1}-1)+\kappa (T,T')(e^{ik_2}-1)\right) +O(N^{-2})\right] ^{N-1}\, \end{aligned}$$

It is elementary to show that this function converges pointwise for all $(k_1,k_2)\in \mathbb {R}^2$ to

11 $$\begin{aligned} \hat{f}(k_1,k_2\mid T) = \exp \left[ \lambda ^-(T)(e^{ik_1}-1) +\lambda ^+(T)(e^{ik_2}-1)\right] \ , \end{aligned}$$

where $\lambda ^-(T)=\sum _{T'}\mathbb {P}(T')\kappa (T',T), \lambda ^+(T)=\sum _{T'}\mathbb {P}(T')\kappa (T,T')$. Finally, we can identify $\hat{f}$ as a product of characteristic functions of Poisson random variables, and therefore the LCT implies the following result.

##### Proposition 2

The in/out degree sequence $(\textrm{d}_v^-,\textrm{d}_v^+)^{(N)}$ of any type *T* bank *v* converges in distribution as $N\rightarrow \infty$ to a bivariate pair $(\textrm{d}_1^-,\textrm{d}_1^+)$ of independent Poisson random variables with parameters $\lambda ^-(T), \lambda ^+(T)$.

Recall that a *finite mixture* of a collection of probability distribution functions is the probability distribution formed by a convex combination. Thus for a finite type space [*M*], the asymptotic *unconditional* bivariate in/out degree distribution of any bank is:

12 $$\begin{aligned} \hat{f}(k_1,k_2)= \sum _{T\in [M]}\mathbb {P}(T) \hat{f}(k_1,k_2\mid T)\ . \end{aligned}$$

which is a finite mixture. Each mixture component has a product Poisson distribution with Poisson parameters

13 $$\begin{aligned} \Bigl (\sum _{T'}\mathbb {P}(T')\kappa (T',T), \sum _{T'}\mathbb {P}(T')\kappa (T,T')\Bigr ) \end{aligned}$$

and the mixing variable is the bank-type *T* with mixing weight $\mathbb {P}(T)$.

#### Distribution of interbank debt

The next result provides the $N\rightarrow \infty$ limit in distribution of the interbank debt $\bar{\textrm{X}}^{(N)}_1=\sum _{w=2}^N\bar{\Omega }_{1w}$ of a typical bank $v=1$.

##### Proposition 3

1. When bank 1 has type *T*, the sequence $\bar{\textrm{X}}^{(N)}_1$ converges in distribution to a random variable $\bar{\textrm{X}}_1$ whose characteristic function is given by:
  14 $$\begin{aligned} \hat{f}_\textrm{X}(k\mid T):= \exp \left[ \sum _{T'}\mathbb {P}(T')\kappa (T,T')(\hat{f}_{\bar{\Omega }}(k\mid T',T)-1)\right] \ . \end{aligned}$$
2. When bank 1 has type *T*, the sequence $\bar{\textrm{X}}^{(N)}_{1\backslash 2}:=\sum _{w=3}^N\bar{\Omega }_{1w}$ converges in distribution to a random variable $\bar{\textrm{X}}_{1\backslash 2}$ with the same characteristic function $\hat{f}_\textrm{X}(k\mid T)$.
3. Let $A\in \mathbb {Z}_+$ be a finite set of positive integers. Then the finite collection of interbank debt random variables $\{\bar{\textrm{X}}_v, v\in A\}$ is independent in the $N\rightarrow \infty$ limit.

##### Proof of Proposition 3

For part (1), the conditional IID (independent, identically distributed) property of the factors of $e^{ik\bar{\textrm{X}}_1}=\prod _{w\ne 1}e^{ikI_{1w}\bar{\Omega }_{1w}}$ implies an exact formula valid for finite *N*:

15 $$\begin{aligned} \hat{f}^{(N)}_\textrm{X}(k\mid T)= & {} \mathbb {E}^{(N)}\left[ \prod _{w\ne 1}\left( 1+ I_{1w}(e^{ik\bar{\Omega }_{1w}}-1)\right) \mid T\right] \nonumber \\= & {} \left( 1+\sum _{T'}\mathbb {P}(T')\frac{\kappa (T,T')}{N-1}\bigl (\hat{f}_{\Omega }(k\mid T,T')-1\bigr )\right) ^{N-1}\ , \end{aligned}$$

where $\hat{f}_\Omega$ is the CF of the distribution given by (8). It is straightforward to show that this sequence of functions converges pointwise for $k\in \mathbb {R}$ to the function $\hat{f}_\textrm{X}(k\mid T)$ given by (14). Equation 14 can be rewritten

16 $$\begin{aligned} \hat{f}_X(k\mid T)=\exp \left[ \int ^\infty _0 [e^{iku}-1]\mu _X(u\mid T)\textrm{d}u\right] \end{aligned}$$

with $\mu _X(u\mid T)= \sum _{T'}\mathbb {P}(T')\ \kappa (T,T')\ \rho _\Omega (u\mid T,T')$, which can be identified as the characteristic function of the positive compound Poisson random variable with finite jump measure $\textrm{d}\mu _X(\cdot \mid T)$ on $\mathbb {R}^+$. Thus the LCT implies the convergence in distribution of the sequence to a compound Poisson random variable $\textrm{X}$. The proof of part (2) also follows by the same method.

For part (3), the joint conditional CF of $\bar{\textrm{X}}_1,\bar{\textrm{X}}_2$ for two banks will be given by

$$\begin{aligned}{} & {} \mathbb {E}^{(N)}[e^{ik_1\bar{\textrm{X}}_1}e^{ik_2\bar{\textrm{X}}_2}\mid T_1,T_2]\\{} & {} \quad =\left( 1+\frac{\kappa (T_1,T_2)}{N-1}\bigl (\hat{f}_{\Omega }(k\mid T_1,T_2)-1\bigr )\right) \left( 1+\frac{\kappa (T_2,T_1)}{N-1}\bigl (\hat{f}_{\Omega }(k\mid T_2,T_1)-1\bigr )\right) \\{} & {} \qquad \quad \times \prod _{w\ne 1,2}\sum _{T'}\mathbb {P}(T')\left( 1+\frac{\kappa (T_1,T')}{N-1}\bigl (\hat{f}_{\Omega }(k\mid T_1,T')-1\bigr )\right) \\{} & {} \qquad \quad \times \left( 1+\frac{\kappa (T_2,T')}{N-1}\bigl (\hat{f}_{\Omega }(k\mid T_2,T')-1\bigr )\right) \end{aligned}$$

The first two factors converge pointwise to 1 and the product is over $N-2$ identical functions that converge pointwise to characteristic functions. Again, LCT applies to yield the stated limit in distribution. Finally, note that the required asymptotic factorization can be proved similarly for the joint conditional CF for any finite collection of banks. $\square$

##### Remark 2

Note that unlike in multitype networks, for a network with only one type ($M=1$), the random variables $\{\bar{\textrm{X}}_v, v\in A\}$ are strictly independent for finite *N*. Thus the proof of part (3) of the Proposition also shows that dependence arises through the conditioning variables $(T_v)$. However, this dependence is weak in the sense it disappears in the $N\rightarrow \infty$ limit.

### Single type networks: the first cascade step

To understand the large *N* asymptotics of the solvency cascade defined for any finite *N* in Sect. 2, we consider the first cascade step in the case of a single type, $M=1$. In this setting, the IRFN skeleton is a directed Erdös-Renyi random graph with a constant mean-degree parameter $\kappa >0$.

Consider for $n=0$ the single shock transmitted from 2 to 1 for two typical banks 1, 2. We write this as $S^{(0)}_{21}=G(X,Y,I_{21}Z)$ where the *shock transmission function* is $G(x,y,z):=z(1-h(y/(x+z))$ with a continuous recovery function *h* as in equation (2). The three random variables $X={\bar{X}}_{2\backslash 1}:=\sum _{w\ne 1, 2} I_{2w}\bar{\Omega }_{2w}, Y:=\min (\Delta ^{(0)}_2,0), Z:= \bar{\Omega }_{21}$ are independent for finite *N*. Since $e^{ikG(X,Y,I_{21} Z)}=1+I_{21}(e^{ikG(X,Y, Z)}-1)$, the characteristic function of $S^{(0)}_{21}$ is given for finite *N* by

17 $$\begin{aligned} \mathbb {E}^{(N)}[e^{ikS^{(0)}_{21}}]=1+\frac{\kappa }{N-1} \int ^0_{-\infty }\ \rho ^{(0)}_\Delta (y) \mathbb {E}^{(N)}[e^{ikG({\bar{X}}_{2\backslash 1},y,\bar{\Omega }_{21})}-1]\ \textrm{d}y \end{aligned}$$

Note also that *G*(*X*, *Y*, *Z*) is a continuous function of a sequence of random vectors (*X*, *Y*, *Z*) that converges in distribution. This implies it converges in distribution as $N\rightarrow \infty$.

The total solvency shock transmitted to bank 1 in step 0 is a sum $S^{(0)}_1:=\sum _{w\ne 1} S^{(0)}_{w1}$ of identical shocks $\{S^{(0)}_{w1}\}_{w\ne 1}$ that, as observed in Remark 2 for the single type IRFN, are independent for any *N*. Therefore,

18 $$\begin{aligned} \mathbb {E}^{(N)}[e^{ikS^{(0)}_{1}}]=\left( \mathbb {E}^{(N)}[e^{ikS^{(0)}_{21}}]\right) ^{N-1} \ . \end{aligned}$$

A simple continuity argument arising from the convergence in distribution of *G*(*X*, *Y*, *Z*), shows that the characteristic function of the *total solvency shock* $S^{(0)}_{1}=\sum _{w\ne 1} S^{(0)}_{w1}$ converges pointwise for $k\in \mathbb {R}$ to

19 $$\begin{aligned} \hat{f}^{(0)}_{S}(k) :=\exp \left( \kappa \int ^0_{-\infty }\ \rho ^{(0)}_\Delta (y) R(k, y)\ \textrm{d}y\right) \ , \end{aligned}$$

where

20 $$\begin{aligned} R(k, y):=\lim _{N\rightarrow \infty }\mathbb {E}^{(N)}[e^{ikG({\bar{X}}_{2\backslash 1},y,\bar{\Omega }_{21})}-1]\ . \end{aligned}$$

Thus the LCT implies that $S^{(0)}_1$ converges in distribution to a random variable with CF $\hat{f}^{(0)}_{S}(k)$. Under regularity conditions such as an $L^2$-condition, one can use the Parseval-Plancherel identity in Fourier analysis and rewrite (19) as

21 $$\begin{aligned} \hat{f}^{(0)}_{S}(k) :=\exp \left( \kappa \int ^\infty _{-\infty }\ \hat{f}^{(0)}_\Delta (k') \hat{R}(k, -k')\ \textrm{d}k'\right) \ , \end{aligned}$$

where

22 $$\begin{aligned} \hat{R}(k, k'):=\frac{1}{2\pi }\int ^0_{-\infty } e^{ik'y} R(k,y)\ \textrm{d}y\ . \end{aligned}$$

The impacted solvency buffer at the end of step 0 is $\Delta ^{(1)}_1=\Delta ^{(0)}_1-S^{(0)}_1$ where $S^{(0)}_1$ and $\Delta ^{(0)}_1$ share no common balance sheet random variables and are therefore independent. From the multiplicative property of characteristic functions of sums of independent random variables, the impacted solvency buffer $\Delta ^{(1)}_1$ has the product characteristic function

23 $$\begin{aligned} \hat{f}^{(1)}_{\Delta }(k) =\hat{f}^{(0)}_{\Delta }(k) \hat{f}^{(0)}_{S}(-k)\ . \end{aligned}$$

We see that step 0 of the single type solvency cascade mapping takes the initial conditional distributional data for the collection $\{I_{vw}, \bar{\Omega }_{vw},\Delta ^{(0)}_v\}$, combined with a conditional independence assumption, and outputs univariate distributional data for the collection $(\Delta ^{(1)}_v)_{v\in [N]}$.

### Solvency cascade: multiple types

There are two reasons the rigorous $N\rightarrow \infty$ argument just presented does not easily generalize to the IRFN framework with $M>1$, or for subsequent cascade steps. First, at step $n=0$ when $M>1$, there is no longer strict conditional independence of the collection of random variables $S^{(0)}_{w1}, w\ne 1$. However, Part 3 of Proposition 3 indicates that the dependence is weak, and goes to zero as $N\rightarrow \infty$. It is therefore highly plausible that the *total solvency shock* $S^{(0)}_1$ converges in distribution to a random variable with CF conditioned on the type $T_1=T$ given by:

24 $$\begin{aligned} \ \hat{f}^{(0)}_{S}(k\mid T) :=\exp \left( \sum _{T'}\mathbb {P}(T')\kappa (T',T)\int ^0_{-\infty }\ \rho ^{(0)}_\Delta (y\mid T') R(k, y\mid T,T')\ \textrm{d}y\right) \end{aligned}$$

where

25 $$\begin{aligned} R(k, y\mid T,T')+1:=\lim _{N\rightarrow \infty }\mathbb {E}^{(N)}[e^{ikG({\bar{X}}_{2\backslash 1},y,\bar{\Omega }_{21})}\mid T_1=T,T_2=T']\ . \end{aligned}$$

Second, even in the single type setting, the collection $\{\Delta ^{(1)}_v\}_{v\in [N]}$ is no longer independent, and thus the proof of the large *N* convergence for $n=0$ does not extend to $n>0$. Nonetheless, the tree independent property of the solvency cascade mechanism proved in Sect. 2.3, combined with the locally treelike property of the skeleton graph, provides justification for conjecturing that the solvency cascade dynamics is given for all *n* by iterates of the mapping from $\Delta ^{(0)}$ to $\Delta ^{(1)}$ defined by (24),(25) and the multitype extension of (23). Thus, we conjecture that for any fixed bank *v* of type *T* and cascade step $n\ge 0$, the sequence of bivariate random variables $(\Delta ^{(n)}_v,S^{(n)}_{v})$ converges in distribution as $N\rightarrow \infty$ to independent random variables with CF given by the following recursive formulas:

**Stochastic Solvency Cascade Mapping: ** Starting from the characteristic functions $\hat{f}_\Delta ^{(0)}(k,T)$ of the initial solvency buffers $\Delta ^{(0)}$, iterate the following two steps for $n=0,1,2,\dots$:

1. Compute the univariate characteristic function $\hat{f}_S^{(n)}(k\mid T)=\mathbb {E}[e^{ikS^{(n)}_{1}}\mid T]$ of the total solvency shock $S^{(n)}_{1}$ using (24) with $\rho _\Delta ^{(0)}$ replaced by $\rho _\Delta ^{(n)}$:
  26 $$\begin{aligned} \hat{f}_S^{(n)}(k\mid T)=\exp \left( \sum _{T'}\mathbb {P}(T')\kappa (T',T)\int ^0_{-\infty }\ \rho ^{(n)}_\Delta (y\mid T') R(k, y\mid T,T')\ \textrm{d}y\right) \ , \end{aligned}$$
  where $R(k, y\mid T,T')$ is given by (25).
2. Compute the univariate distribution of the impacted solvency buffer $\Delta ^{(n+1)}_1=\Delta ^{(0)}_1-S^{(n)}_1$ using the formula (23):
  27 $$\begin{aligned} \hat{f}_\Delta ^{(n+1)}(k\mid T) =\hat{f}_\Delta ^{(0)}(k\mid T) \hat{f}_S^{(n)}(-k\mid T)\ . \end{aligned}$$

Under suitable regularity conditions such as a finite $L^2$ condition, the Parseval-Plancherel identity applied to (26) implies that

28 $$\begin{aligned} \hat{f}_S^{(n)}(k\mid T)=\exp \left( \sum _{T'}\mathbb {P}(T')\kappa (T',T) \int ^\infty _{-\infty } \ \hat{f}_\Delta ^{(n)}(k'\mid T')\ R(k, k'\mid T,T')\ \textrm{d}k'\right) \ , \end{aligned}$$

where

29 $$\begin{aligned} \hat{R}(k, k'\mid T,T'):=\frac{1}{2\pi }\int ^0_{-\infty } e^{ik'y} R(k,y\mid T,T')\ \textrm{d}y\ . \end{aligned}$$

**Cascade Equilibrium: ** When the $N=\infty$ has been specified by computing $\hat{f}_\Delta ^{(0)}(k,T)$ and $\hat{R}(k, k'\mid T,T')$, the dynamic model can be computed by iteration of the main equation:

30 $$\begin{aligned} \hat{f}_\Delta ^{(n+1)}(k\mid T) =\hat{f}_\Delta ^{(0)}(k\mid T) \exp \left( \sum _{T'}\mathbb {P}(T')\kappa (T',T) \int ^\infty _{-\infty } \hat{f}_\Delta ^{(n)}(k'\mid T')\ R(k, k'\mid T,T')\ \textrm{d}k'\right) . \end{aligned}$$

Due to the monotonicity of the underlying finite *N* agent based model, one expects this iteration scheme to converge to a *fixed point* $\hat{f}_\Delta ^{(*)}(k\mid T)$ of the equation:

31 $$\begin{aligned} \hat{f}_\Delta ^{(*)}(k\mid T) =\hat{f}_\Delta ^{(0)}(k\mid T) \exp \left( \sum _{T'}\mathbb {P}(T')\kappa (T',T) \int ^\infty _{-\infty } \hat{f}_\Delta ^{(*)}(k'\mid T')\ R(k, k'\mid T,T')\ \textrm{d}k'\right) . \end{aligned}$$

From $\hat{f}_\Delta ^{(*)}(k\mid T)$, one can easily derive important systemic risk measures, most notably the fraction $p^*(T)$ of eventually defaulted firms of each type *T* given by

32 $$\begin{aligned} p^*(T)=\int _{\mathbb {R}} \hat{f}_\Delta ^{(*)}(k\mid T) \textrm{d}k\ . \end{aligned}$$

One can say that the network is “not resilient” if numerical explorations as in Sect. 5 show that any small initial shock coded into $\hat{f}_\Delta ^{(0)}$ leads to a finite positive value $p^*(T)$.

## Implementation

This section discusses issues to address in implementing the solvency cascade model on IRFNs, and its generalizations, for a large real world network of $\hat{N}$ banks. The first issue is to construct a sequence of IRFNs of size *N* increasing to infinity, that is statistically consistent with the real world pre-crisis financial network when $N=\hat{N}$. Then the statistical model for $N=\infty$ can be subjected to crisis triggers with any distribution of initial shocks $\delta \textrm{B}$, and the resultant solvency cascade analytics developed in Sect. 3 will yield measures of the resilience of the real world network.

### Ideal network data

The type of financial network data available to regulators varies widely from country to country. Here we imagine a country that provides a minimal dataset for $\hat{N}=\sum _{T\in [M]} \hat{N}_T$ banks classified into *M* types labelled by $T\in [M]$, where $\hat{N}_T$ denotes the number of banks of type *T*. Suppose the interconnectivity, exposures and balance sheets of the network have been observed monthly for the past $N_m=12$ months. Bank type will be assumed not to change, but the connectivity and balance sheets will fluctuate over the period. For any of the monthly observations of the network, directed edges are drawn between any ordered pair (*v*, *w*) of banks if the exposure of bank *w* to bank *v* exceeds a specified threshold (a “significant exposure”). Let $\hat{E}=\sum _{T,T'} \hat{E}_{T,T'}$ be the total number of significant exposures in the network identified in the $N_m=12$ month historical database, decomposed into a sum over the bank types involved. For each $T\rightarrow T'$ edge $e\in [\hat{E}_{T,T'}]$ we observe the value $\Omega _e$; For each $v\in [N_m\times \hat{N}_T]$ we also observe samples $\textrm{B}_v$ of the type *T* balance sheets. Our large *N* IRFN will be calibrated to this data.

### Calibrating the large *N* model

The data described above leads to a natural calibration of the pre-trigger IRFN model for any value of $N\ge \hat{N}$ (including $N=\infty$) at any time in the near future. A bank *v* randomly selected from the empirical distribution will have type *T* with probability

$$\begin{aligned} \widehat{\mathbb {P}}(T)=\frac{\hat{N}_T}{\hat{N}}\ . \end{aligned}$$

Conditioned on $T_v=T$, its balance sheet $\textrm{B}_v=[\bar{\textrm{Y}}_v,\bar{\Delta }_v]$ will be drawn from the distribution whose *empirical bivariate characteristic function* is

33 $$\begin{aligned} \hat{f}_ \textrm{B}(u_1,u_2\mid T)=\frac{1}{N_m\times \hat{N}_{T}}\sum _{v=1}^{N_m\times \hat{N}_{T}}e^{iu_1\textrm{Y}_v+iu_2\bar{\Delta }_v}\end{aligned}$$

as a function of $\textbf{u}=(u_1,u_2)\in \mathbb {R}^2$.

A randomly selected pair of banks $e=(v,w), v\ne w$ with types $T,T'$ respectively will have a significant directed exposure, and hence a directed edge, with probability

$$\begin{aligned} \frac{\widehat{\kappa }(T,T')}{\hat{N}-1}=\frac{\hat{E}_{T,T'}}{N_m \hat{N}_T(\hat{N}_{T'}-\delta _{TT'})}\ . \end{aligned}$$

where the matrix $\widehat{\kappa }$ is called the *empirical connection kernel*. Finally, for each ordered pair $T,T'$ we have $\hat{E}_{T,T'}$ observed significant exposures $\Omega _e$ from a *T* bank to a $T'$ bank, leading to the empirical characteristic function

34 $$\begin{aligned} \hat{f}_\Omega (u\mid T,T')=\frac{1}{\hat{E}_{T,T'}}\sum _{e=1}^{\hat{E}_{T,T'}}e^{iu\Omega _e}\ . \end{aligned}$$

The increasing sequence of random IRFN models based on these empirical probability distributions is intended to capture essential aspects of systemic risk in our specific finite real world network. We will need to check that the $N=\infty$ solvency cascade analytics are indeed a reasonably accurate approximation to simulation results for finite *N*.

### Parametrization issues

There are several issues that need to be considered and tested when implementing such a scheme.

1. Network sparsity: What is a reasonable threshold for defining “significant exposures”? There is a tradeoff between increasing the connectivity (reducing sparseness) and the cost of ignoring small exposures: It has been argued that only “large exposures” are important in solvency. Computational burden is not sensitive to the exposure threshold.
2. How many types of banks is reasonable? Again, there is a tradeoff. Taking *M* sufficiently large is important because this is the parameter that determines how realistically the network correlation can be modelled. However, note that the computational burden increases and the power of the statistical estimation decreases with the number of types.
3. How large must *N* be chosen so that the asymptotic analysis is a reasonable approximation? Likely, the accuracy of the large *N* approximation will deteriorate as the number of types increases. How sensitive is the accuracy of the LTI approximation (which relies to some extent on the sparsity of the network) to the choice of exposure threshold?
4. Where can one obtain the data required to calibrate IRFN models? Privacy issues typically imply that exposure data with identified counterparties is not publicly available. Currently such data is often not available even to regulators. So finding real world network data is an impediment to implementing any kind of financial network model.

## Numerical experiments

Efficient computation of the solvency cascade mapping of Sect. 3.4 requires approximate integration of equation (24). We demonstrate how this integral over $\mathbb {R}$ can be approximated by a sum over a lattice of $2\textrm{Nft}$ points $y\in \delta *(\mathbb {Z}\cap [-\textrm{Nft}, \textrm{Nft}-1])$ with a small discretization parameter $\delta$ and large truncation value $\delta \textrm{Nft}$. More efficiently, as developed in Hurd and Gleeson [29], we can use Fast Fourier Transform identities to evaluate (33), (34) for $k,k'$ on the dual lattice $\frac{2\pi }{\delta \textrm{Nft}}\{-\textrm{Nft}+1/2, -\textrm{Nft}+3/2,\dots , \textrm{Nft}-3/2, \textrm{Nft}-1/2\}$.

Without loss of generality, in this section we choose $\textrm{Nft}$ large and measure balance sheets in integer units with $\delta =1$. For any time step $n\ge 0$, and pair of banks *v*, *w* of types $T_v,T_w$, all relevant random variables are assumed to take only a finite number of possible values:

1. The random variables $\Delta ^{(n)}_v,\Omega _{wv},X_{w\setminus v}$ are all required to take $\textrm{Nft}$ possible integer values for a sufficiently large integer $\textrm{Nft}$. We assume $\delta =1$ and that $\Delta ^{(n)}_v$ takes values in $[-\textrm{Nft}/2,\textrm{Nft}/2)\cap \mathbb {Z}$, while $\Omega _{wv},X_{w\setminus v}$ take values in $[0,\textrm{Nft})\cap \mathbb {Z}$.
2. For each possible negative value $\Delta ^{(n)}_v=y\in [-\textrm{Nft}/2,0)\cap \mathbb {Z}$, the transmitted solvency shock $S_{wv}^{(n)}=G(\Omega _{wv},y,X_{w\setminus v})$ is rounded to an integer. For all types $T_v,T_w$, the characteristic function of $S_{wv}^{(n)}$ conditioned on *y* given by (20) is straightforward to approximate for any functional form of *G* by Monte Carlo simulation of size $\textrm{Nmc}$ of pairs $(\Omega _j,X_j)_{j\in [\textrm{Nmc}]}$ for a sufficiently large $\textrm{Nmc}$:
  $$\begin{aligned} R(k,y|T_v,T_w)+1\sim \frac{1}{\textrm{Nmc}}\sum _ {j\in [\textrm{Nmc}]} \exp [ik\ \textrm{round}(G(\Omega _j,y,X_j))] \ . \end{aligned}$$
  This function needs to be computed for *k* taking values on the dual grid $[-\textrm{Nft}/2,\textrm{Nft}/2)\cap \left( \frac{2\pi }{\textrm{Nft}}\mathbb {Z}\right)$.
3. Finally *R* given by (25) for all types $T_v,T_w$ can be treated as a four-dimensional array. $\hat{R}$ given by (29) is a Fast Fourier Transform of *R* in a single dimension.

To ensure that this scheme will generate meaningful results, one should pay attention to several rules of thumb concerning the approximations made. First, we repeat that the choice of unit grid spacing (i.e. taking the discretization parameter $\delta =1$) can be made without loss of generality because the underlying continuum model is invariant under joint rescalings of the collection $\Delta ^{(n)}_v,\Omega _{wv},X_{w\setminus v}$. Second, one needs to choose the number of grid points $\textrm{Nft}$ sufficiently large so that the probabilities $\mathbb {P}(\Delta ^{(n)}_v\le -\textrm{Nft}/2), \mathbb {P}(\Delta ^{(n)}_v\ge \textrm{Nft}/2),\mathbb {P}(X_{w\setminus v}\ge \textrm{Nft})$ are always negligible. Finally, the rounding error for *G* should also be negligible.

These approximation schemes will now be demonstrated in the one-type setting, and will be verified as meaningful by showing how results stabilize for different values of $\textrm{Nft}$ and $\delta$. The extension to many bank types $M>1$ presents no further conceptual difficulty, and should provide tools for full explorations of highly heterogeneous model scenarios.

### Numerical example: a one-type network

To illustrate the above computational procedure, we computed the solvency cascade using the large *N* asymptotics for an IRFN network of a single node type. This means that the DIRG skeleton is a directed Erdös-Renyi configuration graph, with the mean in and out degree parameter $\kappa$. We chose an initial specification of random balance sheets that is similar, but not identical, to the one-type banking model discussed in Hurd [27, Section 4.8]. Unlike that model, exposures $\Omega$ and interbank assets *Z* are random not deterministic, with the detailed parameter choices as follows: (1) All banks have the same non-random initial buffer size $\Delta ^{(0)}=16$; (2) interbank exposures $\bar{\Omega }$ are discrete approximations of gamma-distributed random variables with mean $\mu _\Omega =80/\kappa$ and shape parameter $k=3$; (3) buffers and default shocks take $\textrm{Nft}=2^9$ possible discrete values on the grid with $\delta =0.25$. Note that the mean interbank asset $\mu _Z=80$ and the mean exposure and buffer are each scaled up by a factor 4 relative to the choices in Hurd [27, Section 4.8].

Solvency shocks are assumed to satisfy (3) with the recovery function $h_\lambda (x)=\max \bigl (1,\min (x/\lambda +1,0)\bigr )$. We determined the final fraction of defaults for a range of values for the parameters $\lambda \in (0,1]$ and $\kappa \in (0,10]$. Finally, the stylized financial crisis of each scenario is triggered by an initial shock that changes the buffers of a small fraction $d_0=10^{-5}$ of banks to $\Delta ^{(0)}=-\textrm{Nft}/4$, causing their default.

**Fig. 1:**
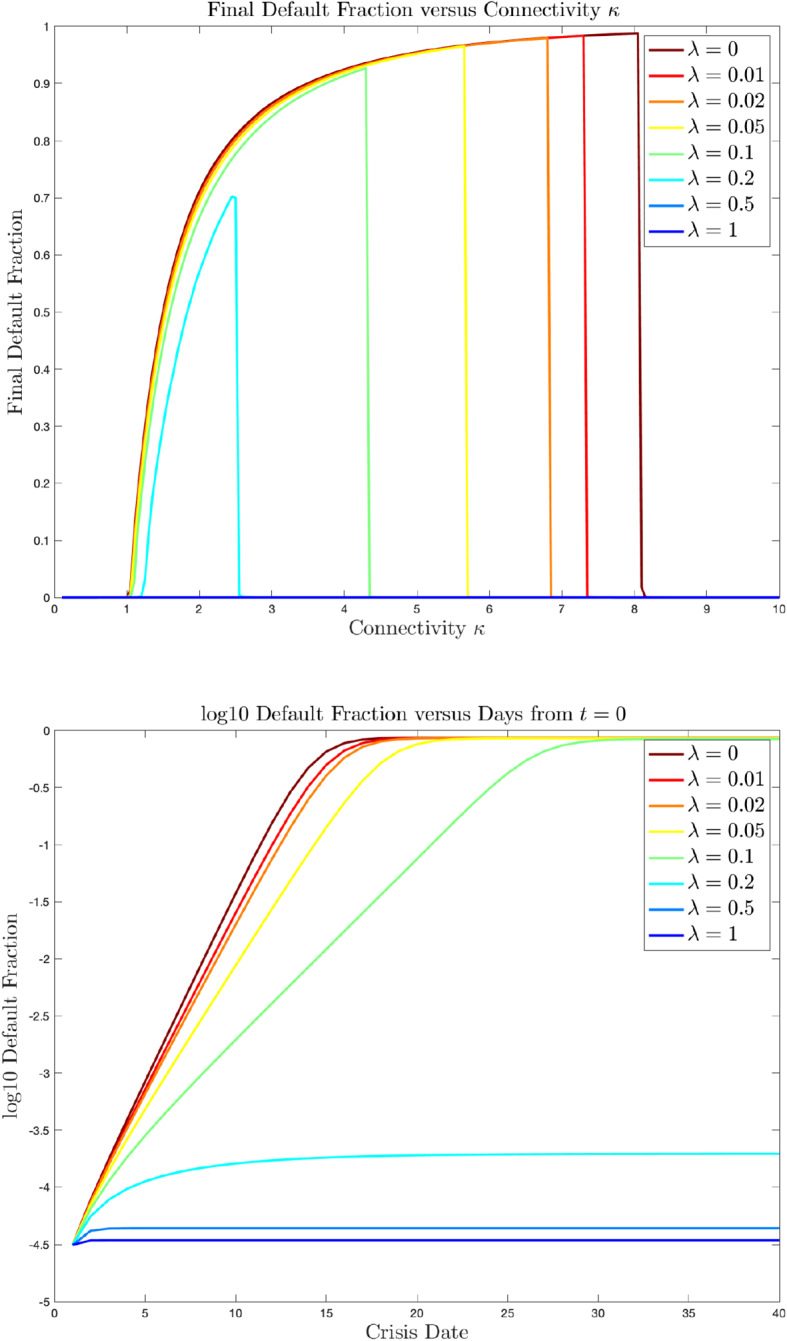
The upper figure shows the final default fraction, as a function of the connectivity parameter $\kappa$. There are clear transitions from “resilience” to “non-resilience” when $\kappa$ exceeds 1. The lower figure shows the time development of the crisis for the value $\kappa =3$. Both figures show the following shock transmission parameter values $\lambda \in \{0,0.01,0.02,0.05,0.1,0.2,0.5,1\}$, where $\lambda =0$ is the zero-recovery case and $\lambda =1$ is the zero-bankruptcy cost case

Figure 1 displays the results of cascade experiments on such networks for a range of possible values of $\lambda$. As expected, the upper figure shows that the final fraction of defaulted banks is monotonically decreasing in the $\lambda$ parameter. Also, across the range of $\kappa$ values, a “resilient” network is observed when bankruptcy costs are small ($\lambda \ge 0.2$). In common with Figure 5.1 of Hurd [27, Section 4.8], the most striking feature of the upper figure is the non-monotonic relationship between the final default fraction and the connectivity parameter $\kappa$, and in particular the sharp downward discontinuity as $\kappa$ exceeds a certain level. This resiliency discontinuity is observed in other network models (see Figure 5.1 of Hurd [27, Section 4.8]), and was given a percolation theoretic explanation by Watts [36] as due to the number of vulnerable nodes being reduced below a critical value as $\kappa$ grows. The lower logarithmic plot shows that in the intermediate non-resilient range of $\kappa$ values, the exponential growth rate observed early in the crisis decreases with $\lambda$ and the system typically reaches its approximate cascade equilibrium (maximal extent) in less than 50 days.

We have experimented further, and found that with the rules of thumb identified in the previous subsection, the computational algorithm leads to conclusions and graphs that are robust to varying implementation parameters $\textrm{Nft},\textrm{Nmc},\delta$. Taking $\textrm{Nft}$ smaller than $2^9$ for the Fast Fourier Transform was found to be insufficient, apparently leading to aliasing errors. Taking $\textrm{Nmc}$ as small as 1000 yielded reliable comparative statics, but with noisy dependence on $\kappa$. The large value $\textrm{Nmc}=100000$ was adequate to generate the smoothly $\kappa$-dependent curves shown here.

### Cascade numerics: algorithmic complexity and runtimes

The numerical implementation of the general cascade mapping for $M\ge 1$ types requires computing the array $\mathcal {R}(k,k'\mid T,T'):=\mathbb {P}(T')\kappa (T',T) \hat{R}(k,k'\mid T,T')$ only once for the entire cascade. For a given FFT size $\textrm{Nft}$, $\mathcal {R}$ can be thought of as a square matrix with $\textrm{Nft}\times M$ rows and columns. Given the solvency cascade kernel ${\mathcal {R}}$, the algorithm for each cascade step maps the $\textrm{Nft}\times M$ dimensional vector $\hat{f}_\Delta ^{(n)}$ to the exponential of a matrix product $\hat{f}_S^{(n)}=\exp [ {\mathcal {R}} * \hat{f}_{\Delta }^{(n)}]$, followed by a Hadamard (element-wise) product $\hat{f}_{\Delta }^{(n+1)}={\textrm{diag}}(\hat{f}_{\Delta }^{(0)})*\hat{f}_S^{(n)}$. Thus the solvency cascade mapping admits a very compact specification in terms of the sequence of conditional characteristic functions, taken as vectors $\hat{f}_{\Delta }^{(n)}:={\hat{\textbf{f}}}^{(n)}\in \mathbb {C}^{\textrm{Nft}\times M}$, namely:

35 $$\begin{aligned} {\hat{\textbf{f}}}^{(n+1)}=\mathcal {C}({\hat{\textbf{f}}}^{(n)}):={\textrm{diag}}({\hat{\textbf{f}}}^{(0)})*\exp [ {\mathcal {R}} *{\hat{\textbf{f}}}^{(n)}]\ .\end{aligned}$$

The nonlinear mapping $\mathcal {C}:\mathbb {C}^{\textrm{Nft}\times M}\rightarrow \mathbb {C}^{\textrm{Nft}\times M}$ is parametrized by ${\mathcal {R}}$ and the solvency buffer distribution ${\hat{\textbf{f}}}^{(0)}$, which, we can also note, must satisfy complex conjugation identities $\overline{ {\mathcal {R}} (k,k'\mid T,T')}= {\mathcal {R}} (-k,-k'\mid T,T')$ and $\overline{f^{(0)}(k\mid T)}=f^{(0)}(-k\mid T)$. A single cascade step is thus of order $O(\textrm{Nft}\times M^2)$ flops plus $\textrm{Nft}\times M$ ordinary exponentiations. In general, a *cascade equilibrium* is a fixed point ${\hat{\textbf{f}}}^*$ of the mapping,

36 $$\begin{aligned} {\hat{\textbf{f}}}^*= {\textrm{diag}}({\hat{\textbf{f}}}^{(0)})*\exp [ {\mathcal {R}} *{\hat{\textbf{f}}}^{*}]\ . \end{aligned}$$

The total run time for the numerical experiment of Sect. 5.1, implemented in Matlab on a desktop computer, with $\textrm{Nmc}=100000$, $\textrm{Nft}=2^9$, $t_{max}=40$ days, and eight values of $\lambda$, was about 1400 seconds.

## Open questions

This article introduces IRFNs as a flexible, scalable analytical tool for understanding aspects of systemic risk. A comparable inhomogeneous random network framework applied to infectious disease modelling in Hurd [26] shows the robustness and adaptability of this type of mathematics. As observed repeatedly in this article, the large *N* stochastic solvency cascade mapping stated in Sect. 3.4 remains conjectural. Undoubtably the most important unfinished task stemming from this article is to provide a rigorous derivation of the formulas provided in Sect. 3.4: Success in this direction will be an important theoretical contribution to network science. We end with a brief discussion of three other open questions to investigate.

The first question asks about the computational feasibility and accuracy for models of this type. In Sect. 5 we have presented preliminary but promising results in this direction. By its nature, the IRFN modelling framework scales to very complex specifications: our hope is that the large *N* asymptotic formulas will prove to be an effective tool that accurately reflects the systemic resilience of finite size complex networks.

A second line of inquiry focusses on calibrating IRFN models of this type to real world financial systems. Here the critical issue is the availability of data along the lines discussed in Sects. 4.1 and 4.2. Where a suitable representation of a real world network can be found, it will then be of interest to investigate the multiple dimensions of vulnerability exhibited by the calibrated solvency cascade model.

A third open question is how network models can be used as effective tools to explore and understand further systemic risk effects. Examples of important effects worthy of study include: the impact of exceptional nodes such as a central bank or central clearing party; overlapping contagion channels such as funding liquidity and solvency; more fine-grained balance sheets and exposures; exploring different assumptions on resolution of failed banks; more complex strategic behaviour of banks; more diverse types of nodes such as funds, firms and other economic entities.
